# ROS Defense Systems and Terminal Oxidases in Bacteria

**DOI:** 10.3390/antiox10060839

**Published:** 2021-05-24

**Authors:** Vitaliy B. Borisov, Sergey A. Siletsky, Martina R. Nastasi, Elena Forte

**Affiliations:** 1 Belozersky Institute of Physico-Chemical Biology, Lomonosov Moscow State University, Leninskie Gory, 119991 Moscow, Russia; siletsky@belozersky.msu.ru; 2 Department of Biochemical Sciences, Sapienza University of Rome, Piazzale Aldo Moro, 5, 00185 Rome, Italy; nastasi.1948272@studenti.uniroma1.it

**Keywords:** bacteria, redox enzymes, terminal oxidases, reactive oxygen species, oxidative stress

## Abstract

Reactive oxygen species (ROS) comprise the superoxide anion (O_2_^•−^), hydrogen peroxide (H_2_O_2_), hydroxyl radical (^•^OH), and singlet oxygen (^1^O_2_). ROS can damage a variety of macromolecules, including DNA, RNA, proteins, and lipids, and compromise cell viability. To prevent or reduce ROS-induced oxidative stress, bacteria utilize different ROS defense mechanisms, of which ROS scavenging enzymes, such as superoxide dismutases, catalases, and peroxidases, are the best characterized. Recently, evidence has been accumulating that some of the terminal oxidases in bacterial respiratory chains may also play a protective role against ROS. The present review covers this role of terminal oxidases in light of recent findings.

## 1. Introduction

Reactive oxygen species (ROS) are partially reduced oxygen derivatives. They include the superoxide anion (O_2_^•−^), hydrogen peroxide (H_2_O_2_), hydroxyl radical (^•^OH), and singlet oxygen (^1^O_2_). ROS can be produced within the cell as an unavoidable consequence of bacterial metabolism or derived from the environment. ROS are generated by the host innate immune system in response to bacterial colonization. Invading pathogens are recognized by pattern recognition receptors located on the surface of a phagocyte. As a result, in the course of phagocytosis, the production of ROS and reactive nitrogen species (RNS) is triggered to generate bactericidal oxidative stress [1]. O_2_^•−^ is generated by the phagocyte NADPH oxidase. O_2_^•−^ can then undergo dismutation to form H_2_O_2_ spontaneously or enzymatically by superoxide dismutase. H_2_O_2_ is also generated by many microorganisms at concentrations sufficient to kill their nearby competitors. For instance, arginine-replete *Streptococcus gordonii* monocultures can maintain H_2_O_2_ concentrations within 20–30 μM throughout exponential growth [2]. In exponentially growing *Escherichia coli* (*E. coli*) cells, H_2_O_2_ production was estimated to occur at rates of 9–22 μM/s using strains lacking intracellular scavenging enzymes and grown on a variety of growth substrates [3]. H_2_O_2_ permeates freely across bacterial membranes and can react with Fe^2+^, producing a very powerful oxidant through this Fenton reaction, ^•^OH. One more extremely dangerous ROS, ^1^O_2_, can be generated by endogenous photosensitizers, such as flavins, quinones, porphyrins, and rhodopsins [4]. All these ROS, particularly ^•^OH and ^1^O_2_, can damage bacterial DNA, RNA, proteins, and lipids. To protect themselves against ROS-induced oxidative stress, bacteria utilize different ROS defense mechanisms, of which the enzymatic ROS scavengers, such as superoxide dismutases, catalases, and peroxidases are the best characterized [1,5]. Superoxide dismutases catalyze the dismutation of 2O_2_^•−^ into H_2_O_2_ and O_2_ with the participation of 2H^+^ as co-substrate. The decomposition of H_2_O_2_ is usually conducted by catalases or peroxidases. Catalases catalyze the disproportionation of 2H_2_O_2_ into 2H_2_O and O_2_. Peroxidases catalyze the reduction of H_2_O_2_ (and/or organic hydroperoxides) by a wide variety of organic and inorganic substrates that serve as electron donor. In the case of *E. coli*, the most studied species of bacteria, the following enzymes are used to degrade H_2_O_2_ in vivo: the KatG and KatE catalases [6], the NADH peroxidase AhpCF [7], and the periplasmic cytochrome *c* peroxidase YhjA (also denoted as Ccp) that possesses quinol peroxidase activity [8,9].

Recently, evidence has been accumulated indicating that some of the enzymatic complexes of the terminal segment of the O_2_-dependent respiratory chains, terminal oxidases, may also contribute to ROS defense mechanisms in bacteria. These enzymes catalyze the four-electron reduction of O_2_ to 2H_2_O using quinol or cytochrome *c* as the electron donor [10,11,12,13,14,15]. The membrane-embedded terminal oxidases include the superfamily of heme-copper oxidases [13,14,16,17,18,19,20,21,22,23,24] and the family of copper-lacking *bd*-type oxidases (cytochrome *bd*) [11,25,26,27,28,29]. All these oxidases couple the catalytic redox reaction to the generation of a proton motive force [30,31,32]. Unlike cytochrome *bd* [33,34,35], the heme-copper oxidases create the proton motive force not only due to the transfer of protons and electrons to the catalytic site from different sides of the membrane but also due to a unique mechanism of the proton pumping [36,37]. This is a likely reason why the proton to electron stoichiometry (characteristic of the bioenergetic efficiency) of the heme-copper oxidases is 1.5-2 times higher than that of cytochrome *bd* [30,38]. Heme-copper oxidases are divided into families A, B and C based on the constituents of their proton channels [39,40,41]. Cytochrome *bd*, in turn, can be classified into two subfamilies, S and L, based on the size of a hydrophilic region between transmembrane helices 6 and 7 of subunit I, denoted as the Q-loop [42,43]. A heme-copper oxidase usually carries three or four redox centers depending on whether it is a quinol oxidase or cytochrome *c* oxidase (COX). In addition to the electron entry subunit that carries a binuclear Cu_A_ center, some COXs (*caa*_3_, *cbb*_3_) have an additional domain, the substrate cytochrome *c* [44,45,46]. A distinctive feature of the heme-copper oxidase superfamily is an active site, called the binuclear center (BNC), which consists of a high-spin heme (*a*_3_, *b*_3_, or *o*_3_) and a copper ion (Cu_B_) close to the heme-iron. In the binuclear center, O_2_ is reduced to two molecules of H_2_O. All cytochrome *bds* known to date are quinol (ubiquinol or menaquinol) oxidases. A typical cytochrome *bd* has three redox centers, hemes *b*_558_, *b*_595_, and *d* but no copper. The high-spin heme *d* is the site in which the oxygen chemistry takes place. Sometimes heme *d* is replaced by heme *b* [47]. Cytochrome *bd* usually reveals a much higher affinity for O_2_ than heme-copper oxidases [48,49,50,51].

While the main role of most heme-copper oxidases in microbial metabolism is to conserve energy, cytochrome *bd* appears to serve other important functions in bacteria [52,53,54,55,56]. The *bd*-type oxidases were reported to endow bacteria with resistance to nitric oxide (NO) [57,58,59,60,61,62,63,64,65,66], peroxynitrite [53,67], sulfide [68,69,70,71], ammonia [72], cyanide [68,73,74]. This is probably the reason why cytochrome *bd* is so common in pathogenic bacteria [75]. The absence of these enzymes in eukaryotes makes them very attractive as potential targets for new antibacterial drugs [76,77,78,79,80,81].

In this review, we discuss the contribution of the *bd*-type oxidases and other terminal oxidases to oxidative stress defense mechanisms in bacteria in light of recent findings.

## 2. The *bd*-Type Oxidases by Fast O_2_ Scavenging Protect O_2_-Labile Enzymes from Oxidative Inactivation and Reduce Intracellular ROS Levels

**Figure 1:**
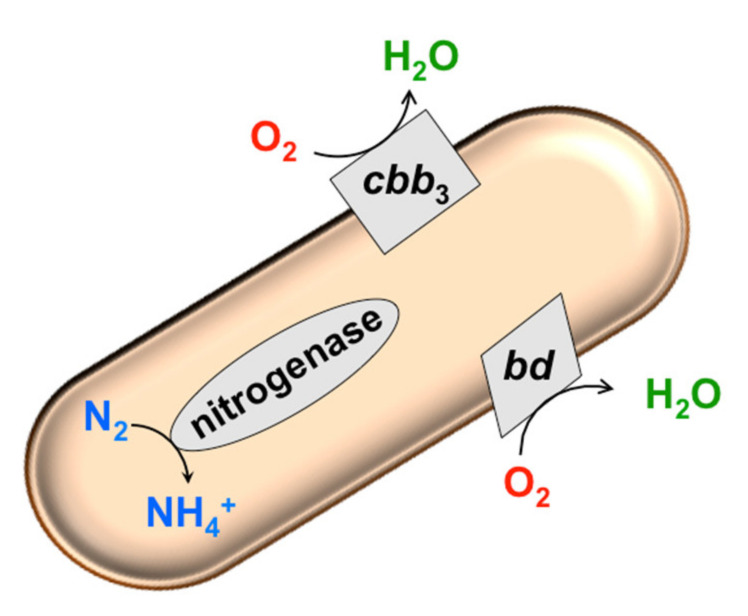
Cytochrome *bd* and cytochrome *cbb*_3_ protect O_2_-labile nitrogenase from oxidative inactivation.

Possibly due to the lack of proton-pumping machinery, cytochrome *bd* generally consumes O_2_ much more rapidly than heme-copper oxidases. In *E. coli* and *Azotobacter vinelandii*, the bimolecular rate constant for O_2_ reaction with the *bd* enzyme approaches diffusion control [82]. This trait allows the *bd* oxidase to play a crucial role in “respiratory protection” of nitrogenase, the O_2_-labile N_2_-fixing enzyme complex, even under aerobic conditions [83] (Figure 1). The prevention of O_2_ inhibition of nitrogenase activity by cytochrome *bd* was shown in *Azorhizobium caulinodans* [84], *A. vinelandii* [83], *Klebsiella pneumoniae* [85]. This is in agreement with the fact that mutant strains lacking cytochrome *bd* are not able to fix nitrogen in the air [86]. Due to the presence of the *bd* enzyme, some bacteria classified as strict anaerobes, e.g., *Bacteroides fragilis* [87] and *Desulfovibrio gigas* [88,89], can survive at low O_2_ concentrations. In this case, apart from protection against the deleterious effects of O_2_, cytochrome *bd* provides the bacteria with the proton motive force to drive ATP synthesis and dissipates excess reducing equivalents via the O_2_-dependent respiratory chain. Consistently, in the anoxygenic phototroph *Rubrivivax gelatinosus*, the *bd* oxidase is used to reduce the environmental O_2_ pressure [90]. This expands the physiological range of ambient O_2_ tensions for this bacterium under which photosynthesis can be initiated. In *E. coli*, a facultative anaerobic bacterium, cytochrome *bd* inhibits the production of intracellular H_2_O_2_ by reduced fumarate reductase. This is observed when anaerobic cultures of an *E. coli* strain devoid of canonical H_2_O_2_-scavenging enzymes KatG, KatE, and AhpCF are abruptly aerated [91]. An underlying mechanism for this phenomenon upon aeration is likely the action of cytochrome *bd* as an electron sink. The *bd* enzyme pulls electrons away from fumarate reductase via the quinone pool. As a consequence, the rate at which fumarate reductase generates H_2_O_2_ decreases [91].

## 3. Bacterial Mutants Devoid of Cytochrome *bd* Show Higher Sensitivity to H_2_O_2_. Cytochrome *bd* Expression Increases in the Presence of H_2_O_2_

Cytochrome *bd* plays a role in protecting bacterial cells against oxidative stress caused by H_2_O_2_. *E. coli* mutant cells devoid of cytochrome *bd*-I (encoded by the *cydAB* operon) are extremely sensitive to H_2_O_2_ exposure [92,93,94]. Consistently, expression of cytochrome *bd*-I in *E. coli* K-12 increases in the presence of external H_2_O_2_ [94]. In uropathogenic *E. coli*, the doubling time of strains lacking either cytochrome *bd*-I or cytochrome *bd*-II (encoded by the *cyxAB* operon) increases considerably following treatment with 1 mM H_2_O_2_ [66]. Such a protective function of the *bd* enzyme is not limited to *E. coli* strains. In the case of *A. vinelandii* cells, 0.15 mM H_2_O_2_ appeared to be more toxic to the mutant strain devoid of the *bd* oxidase than to the wild-type strain [95]. The mutant strain of the sulfate-reducing bacterium *Alishewanella* sp. WH16-1, deficient in cytochrome *bd*, is also more sensitive to H_2_O_2_ than the wild type and complemented strain [96]. Similarly, *Brucella abortus* mutants lacking the *bd* oxidase activity show higher sensitivity to added H_2_O_2_ [97]. This sensitivity is reversed after the introduction of a plasmid (pSEK102) that contains a copy of the *cydAB* operon. Overexpression of superoxide dismutase and catalase can also alleviate the loss of cytochrome *bd* [97], emphasizing that the antioxidant properties of these enzymes are of similar importance. In *Porphyromonas gingivalis* involved in the pathogenesis of periodontitis, the absence of the *bd* oxidase leads to an increase in the susceptibility of exponentially growing bacteria to 0.5 mM H_2_O_2_ [98]. The complementation of the *P. gingivalis* mutant with the native *cydAB* genes partially restores the resistance of the cells to H_2_O_2_ treatment. Small et al. [99] reported the catalase-independent hyper-resistance to H_2_O_2_ in *Mycobacterium tuberculosis* cells overexpressing the *bd* enzyme. The hypersensitivity of the *cydAB* mutants to exogenous H_2_O_2_ was also documented for *Mycobacterium smegmatis* [100]. Consistently, in *Staphylococcus aureus*, the *cydAB* genes are strongly (by 8-9-fold) induced upon 20 min of exposure to H_2_O_2_ [101]. Altogether, these data suggest that at least in a few bacteria, including pathogenic strains, cytochrome *bd* contributes to mechanisms that provide bacterial defense against H_2_O_2_-induced oxidative damage.

## 4. Catalase-Like Activity of Cytochrome *bd*

**Figure 2:**
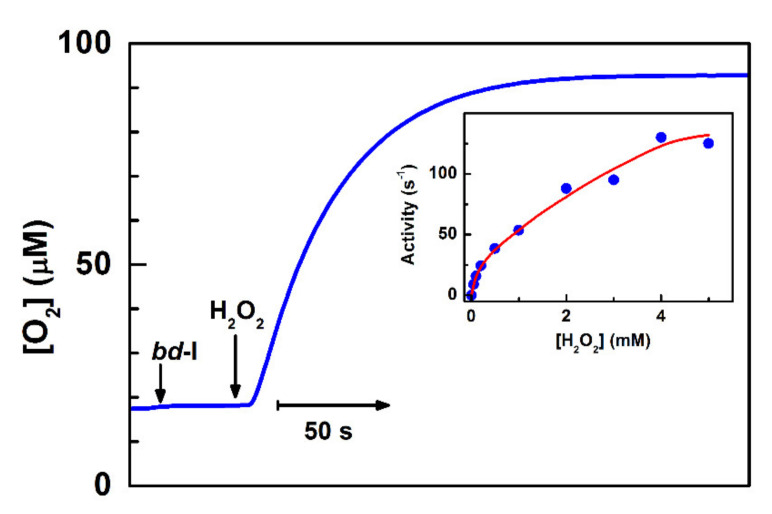
Catalase-like activity of the isolated as-prepared cytochrome *bd*-I from *Escherichia coli* (*E. coli*). *Main panel:* O_2_ formation induced by addition of 0.1 mM H_2_O_2_ to the oxidase. *Inset:* Dependence of the rate of O_2_ formation on H_2_O_2_ concentration. Adapted from [102].

**Figure 3:**
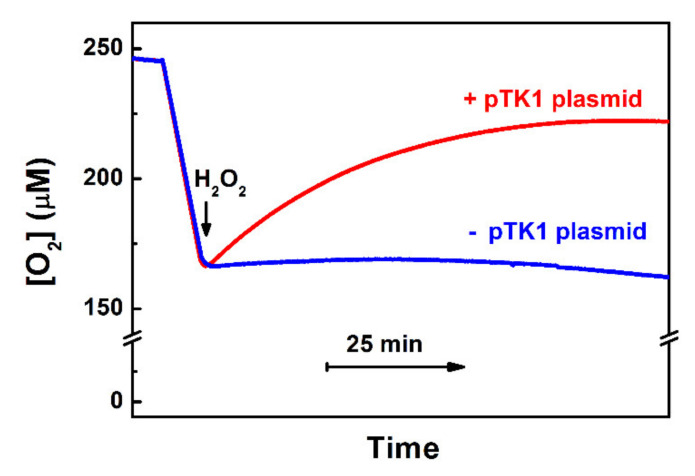
Catalase-like activity of catalase-deficient *E. coli* UM2 cells overexpressing cytochrome *bd*-I. Shown is the change in O_2_ concentration after the addition of 0.235 mM H_2_O_2_ to respiring cells in which the enzyme is either overexpressed (+pTK1 plasmid that carries the *cydAB* operon) or not (−pTK1 plasmid). Adapted from [102].

Apart from the above-described ways by which cytochrome *bd* can decrease intracellular ROS levels indirectly, the enzyme was reported to be able to metabolize H_2_O_2_ directly. Borisov et al. [102] reported that the addition of Н_2_О_2_ to the isolated as-prepared cytochrome *bd*-I from *E. coli* results in the О_2_ evolution in a sealed respirometry chamber (Figure 2, main panel). The observed rate of О_2_ evolution is proportional to the enzyme concentration. The reaction rate also increases linearly with the Н_2_О_2_ concentration, up to 0.2–0.5 mM of the reactant. At higher [Н_2_О_2_], however, the dependence exhibits somewhat saturation behavior (Figure 2, inset), which may be due to partial inactivation of cytochrome *bd*-I by ROS. In this reaction, there is the evolution of approximately one О_2_ molecule per every two Н_2_О_2_ molecules decomposed, implying the catalase-like reaction mechanism. A series of experiments show that the reaction is indeed associated with the *bd*-I enzyme [102]. After the thermal inactivation of cytochrome *bd*-I, the О_2_ evolution is no longer detected. Hence, the possible presence of trace amounts of adventitious transition metals cannot be the reason for the observed О_2_ evolution. The addition of NO, even at a concentration of 20 μM, does not affect the rate of O_2_ formation. At the same time, NO was reported to inhibit bona fide catalase with *K*_i_ of ~0.18 µM [103]. Furthermore, if the *bd*-I enzyme is reduced completely with dithiothreitol (DTT) and 2,3-dimethoxy-5-methyl-6-(3-methyl-2-butenyl)-1,4-benzoquinone (Q_1_), the catalase-like activity is lacking. However, if bona fide catalase is then added to the chamber, the О_2_ evolution resumes. It is hard to imagine that a contaminant catalase, if present, would be redox (DTT/Q_1_)-sensitive, especially as many catalases are not reducible with as strong a reducing agent as dithionite [104], even in the presence of a mediator [105]. Thus, the latter two findings suggest that the isolated *untagged* cytochrome *bd*-I, rather than a potential presence of a native catalase as a contaminant, is responsible for the observed activity. It should be noted that this conclusion is not consistent with the data of Al-Attar et al. [106]. They reported that the isolated *His*_6_*-tagged* cytochrome *bd*-I of *E. coli* does not perform a catalase-like activity as the addition of 1 mM Н_2_О_2_ to the enzyme does not lead to О_2_ generation [106]. Al-Attar et al. proposed that the catalase-like activity of cytochrome *bd*-I shown by Borisov et al. [102] might be due to impurities that include an unknown membrane-associated catalase. However, such an activity is also detected in vivo [102]. Substantial rates of О_2_ production are observed if H_2_O_2_ is added to respiring *E. coli* UM2 cells devoid of KatE and KatG but overexpressing the *bd*-I enzyme (Figure 3, red line). If cytochrome *bd*-I is not overexpressed, the reaction is not seen (Figure 3, blue line). This can only happen if “an unknown membrane-associated catalase” in the cells is cytochrome *bd*-I. This discrepancy may be attributed to the differences between the protein forms (untagged vs. hexahistidine-tagged) or other experimental conditions used for protein expression and purification that Al-Attar et al. also do not exclude. Additional work is needed to resolve the controversy.

**Figure 4:**
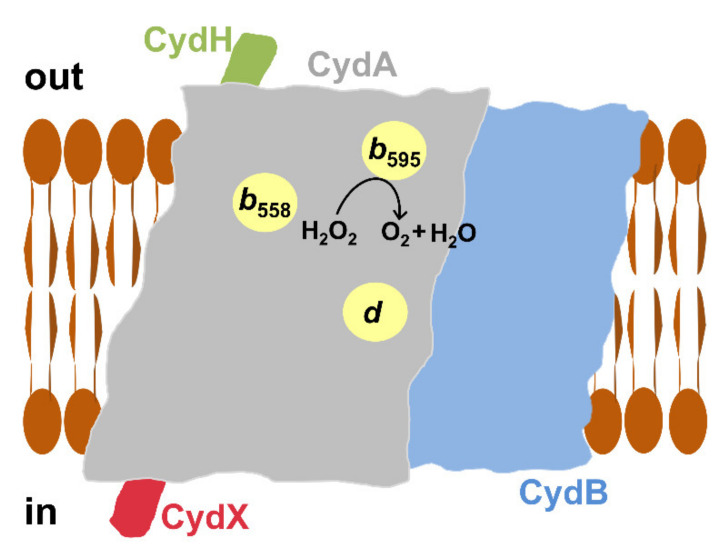
Proposed catalase-like activity of cytochrome *bd*-I and cytochrome *bd*-II from *E. coli*. Shown is the scheme for *bd*-type enzyme arrangement in the *E. coli* membrane bilayer based on the solved *bd*-I structure [28,29]. The oxidase consists of four different subunits, CydA, CydB, CydX, and CydH. CydA carries three hemes, *b*_558_, *b*_595_, and *d*.

The molecular mechanism underlying the catalase-like activity of cytochrome *bd*-I remains unclear. To try to identify the enzyme site responsible for the observed reaction, a few compounds targeting different sites were tested [102]. Antimycin A (167 µM), which inhibits the *bd*-I oxidase via interaction with the quinol binding site [107], does not affect the О_2_ evolution. Consistently, 250 µM oxidized Q_1_ also does not inhibit the reaction. Hence, the quinol binding site does not participate in the activity. Similarly, the rate of О_2_ formation is not affected by 20 µM *N*-ethylmaleimide, a small organic electrophile that blocks cysteine thiols through covalent modification [108]. This suggests that the enzyme thiol groups are also not involved in the reaction. Neither 20 μM NO nor 2 μM CO inhibits the О_2_ evolution. The canonical О_2_ reductase activity of cytochrome *bd*-I was reported to be blocked by NO and carbon monoxide (CO) with *K*_i_ of 100 [57] and 40 nM [109], respectively. Since both NO and CO do this through binding to heme *d*, the participation of this heme in the catalase-like activity is not very likely. This conclusion is also supported by the fact that the catalase-like and the heme *d*-based О_2_ reductase activities do not seem to compete with each other. The reaction is also insensitive to its product, О_2_, as the rates of О_2_ evolution at 3 and 255 µM О_2_ are virtually identical. Notwithstanding this, two small molecules were found to effectively inhibit the catalase-like activity, cyanide and azide. These ligands are known to block heme-containing enzymes by targeting ferric heme-iron. The О_2_ evolution is inhibited by cyanide with a *K*_i_ of 2.5 µM. Consistently, 100 µM azide inhibits the activity almost completely—by 98%. The catalase-like activity appeared to be approximately three orders of magnitude more sensitive to these ligands than the heme *d*-based О_2_ reductase one. This indicates that a heme, but not heme *d*, is involved in the reaction. The site at which the catalase-like chemistry occurs could be heme *b*_595_ (Figure 4). It is pentacoordinate high-spin and therefore can potentially bind an external ligand, such as H_2_O_2_ [110]. It also cannot be ruled out that this catalytic role is played by heme *b*_558_. Although this is a hexacoordinate low-spin heme, the bond between its sixth axial ligand Met393 and the iron ion is weak and can be replaced with a stronger external ligand [111]. Surprisingly, the addition of cyanide to the as-prepared cytochrome *bd*-I at a concentration (50 µM) that fully inhibits the catalase-like activity induces small absorption changes as if the ligand reacts with only some small population of heme *b*. If this is the case, only a fraction of the enzyme (2–4%) is involved in the reaction but with an apparent turnover number greater than 3000 s^−1^ [102]. The catalase-like activity of cytochrome *bd*-I could be induced in vivo in response to the oxidative stress by post-translational protein modification, proteolysis, protein truncation in the translation process, or interaction of the enzyme with other cellular components.

**Figure 5:**
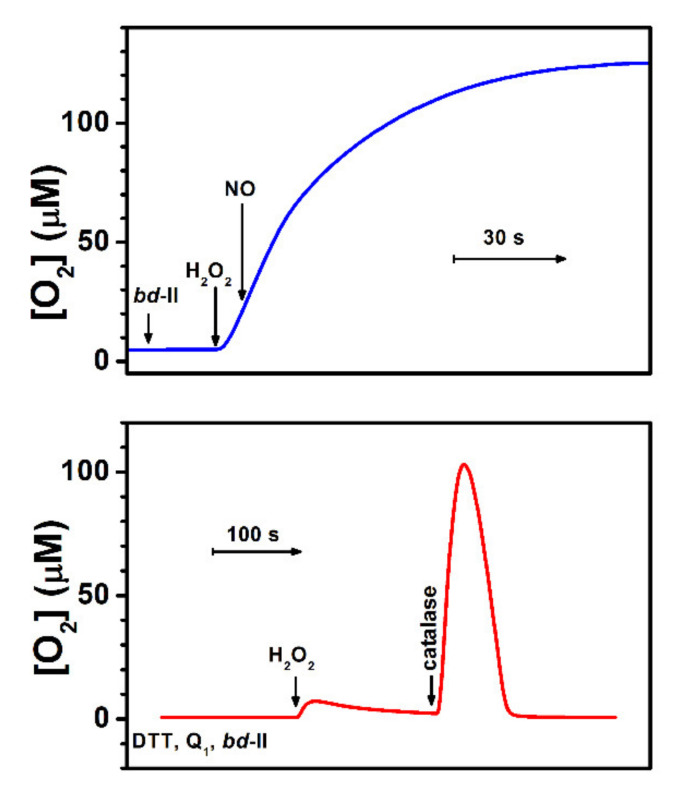
Catalase-like activity of the isolated cytochrome *bd*-II from *E. coli*. *Top panel:* The addition of 20 µM NO does not affect О_2_ evolution induced by the addition of 0.2 mM H_2_O_2_ to the as-prepared enzyme (50 nM). *Bottom panel:* О_2_ evolution is lacking if, before the addition of 1.32 mM H_2_O_2_, all O_2_ is consumed and cytochrome *bd*-II (12.8 nM) is converted into the fully reduced state by 10 mM DTT and 250 µM Q_1_. Subsequent addition of bona fide bovine catalase (2 µg/ml) restores the reaction. Cytochrome *bd*-II was isolated from *E. coli* strain MB37 as described [38]. Changes in O_2_ concentration were recorded using a high-resolution respirometer (Oxygraph-2k, Oroboros Instruments). Assays were performed at 25 °C in 50 mM Na/phosphate buffer (pH 7.0) containing 0.1 mM ethylenediaminetetraacetate (EDTA), supplemented with 0.02% dodecyl-β-D-maltoside.

Preparations of untagged cytochrome *bd*-II isolated from *E. coli* also show high catalase-like activity. Similar to cytochrome *bd*-I, NO at a high concentration (20 µM) does not affect the activity (Figure 5, top panel). The observed О_2_ evolution is also susceptible to the *bd*-II enzyme redox-state. When cytochrome *bd*-II is converted into the fully reduced state following the consumption of all O_2_ in turnover with excess DTT and Q_1_, the H_2_O_2_-induced catalase-like activity is no longer observed. However, if a bona fide catalase is subsequently added, the reaction proceeds (Figure 5, bottom panel). Further studies will show how this discovered activity of cytochrome *bd*-II (Figure 4) contributes to the bacterial defense mechanisms against oxidative stress in vivo. In this regard, a very recent report by Chanin et al. [112] on the role of cytochrome *bd*-II-mediated aerobic respiration of *E. coli* during intestinal inflammation deserves attention. In the course of the inflammatory process, the host produces antimicrobial products including O_2_^•−^ to impede bacterial growth. The O_2_^•−^ molecules generated by the Nox1 NADPH oxidase undergo rapid dismutation to H_2_O_2_ and O_2_ by superoxide dismutase. Using chemical and genetic murine models of noninfectious colitis, Chanin et al. showed that cytochrome *bd*-II provides a fitness advantage for *E. coli* during anaerobic growth in the presence of H_2_O_2_ in the inflamed murine intestine. In the absence of *Nox1*, this fitness advantage is ablated. To do this, the *bd*-II enzyme may use H_2_O_2_ or its breakdown product O_2_ generated by the catalases KatE and KatG, as the substrate. It turned out that in the absence of KatE and KatG, at 5 µM H_2_O_2_, the wild-type strain outcompetes the mutant strain devoid of cytochrome *bd*-II. For this reason, Chanin et al. concluded that O_2_ produced by catalase-mediated degradation of H_2_O_2_ serves as the terminal electron acceptor for the *bd*-II oxidase [112]. However, given the observed catalase-like activity of cytochrome *bd*-II (Figure 5, top panel), the possibility that at higher H_2_O_2_ concentrations, cytochrome *bd*-II could also metabolize H_2_O_2_ in vivo, contributing to the O_2_ pool formation in the inflamed gut, cannot be excluded. Whatever the exact mechanism is, detoxification of the host-derived ROS through cytochrome *bd*-II allows *E. coli* to respire in an otherwise anaerobic environment, promoting bacterial outgrowth [112].

Reduced catalase-like activity was determined in cell-free extracts of *A. vinelandii* when comparing the mutant strain MK5 devoid of the *bd* oxidase and the wild-type strain UW136 [95]. In *Alishewanella* sp. WH16-1, cytochrome *bd* is also suggested to catalyze the decomposition of H_2_O_2_ via the catalase-like reaction (see Figure 7 in [96]). A dramatic increase in resistance of *M. tuberculosis* to Н_2_О_2_ upon the overexpression of cytochrome *bd* reported by Small et al. [99] could be explained, at least in part, by the ability of the *bd* oxidase to perform the catalase-like reaction [113].

## 5. Peroxidase-Like Activity of Cytochrome *bd*

Borisov et al. reported [114] that the isolated untagged cytochrome *bd*-I from *E. coli* displays a peroxidase-like activity. Under aerobic conditions, the enzyme can catalyze the oxidation of guaiacol (*o*-methoxyphenol), benzohydroquinone, ferrocene, and ferrocyanide upon the addition of H_2_O_2_. Using guaiacol as the electron donor, the effect of a few inhibitors of the O_2_ reductase activity of cytochrome *bd*-I on the peroxidase-like activity was studied. It turned out that 2-*n*-heptyl 4-hydroxyquinoline-N-oxide (HQNO), pentachlorophenol, and cyanide inhibit both activities at similar concentrations [114]. Based on the inhibitory analysis, it was concluded that guaiacol binds and donates electrons to the quinol binding site of cytochrome *bd*-I. The electrons are then transferred to the heme *d* site at which H_2_O_2_ is bound and reduced to 2H_2_O. Although an apparent turnover number for the guaiacol peroxidation reaction is as low as about 4 s^−1^, it was suggested [53] that this value could be much higher in vivo where the natural quinol is used as the electron donor.

**Figure 6:**
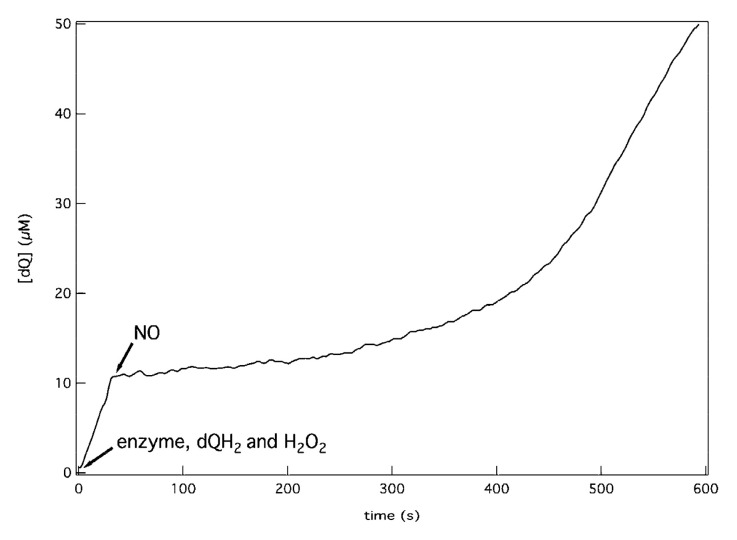
Inhibition of decyl-ubiquinol (dQH_2_) peroxidase activity of the isolated cytochrome *bd*-I from *E. coli* by NO. The reaction is monitored spectrophotometrically under anaerobic conditions. The addition of 6 μM NO promptly inhibits the enzymatic oxidation of 0.2 mM dQH_2_ by 10 mM H_2_O_2_. The inhibition is reversible as the activity gradually resumes due to the disappearance of NO. The latter is probably due to the reaction between NO and dQH_2_. Reprinted from [106].

**Figure 7:**
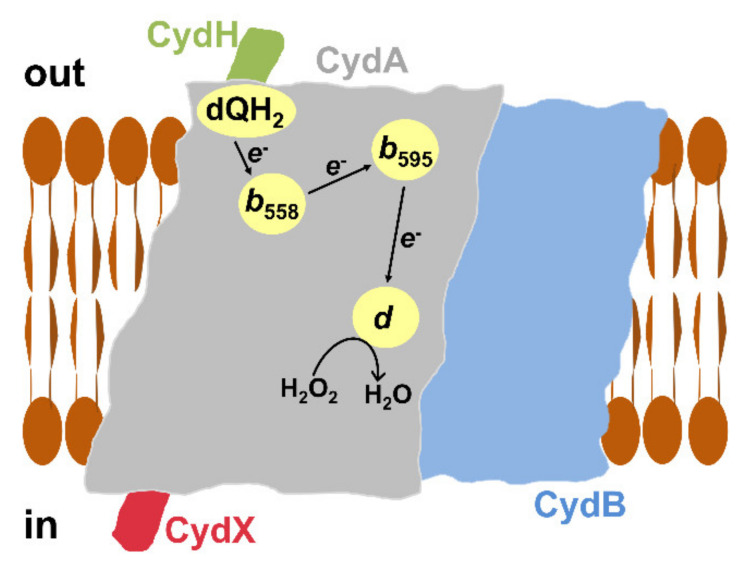
Proposed peroxidase-like activity of cytochrome *bd*-I from *E. coli*. Shown is the scheme for the enzyme arrangement in the *E. coli* membrane bilayer based on the solved *bd*-I structure [28,29]. The oxidase consists of four different subunits, CydA, CydB, CydX, and CydH. CydA carries three hemes (*b*_558_, *b*_595_, *d*) and the quinol binding site at which the electron donor dQH_2_) is likely bound.

Consistent with this, Al-Attar et al. later reported [106] that, under anaerobic conditions, the isolated *His*_6_*-tagged* cytochrome *bd*-I of *E. coli* shows significant peroxidase-like activity. As the electron donor, decyl-ubiquinol (dQH_2_) was used and the oxidation of dQH_2_ by H_2_O_2_ was measured spectrophotometrically by monitoring the absorption change at 260 nm. The average dQH_2_/H_2_O_2_ ratio appeared to be 1.05 ± 0.19, which is consistent with the peroxidase reaction mechanism. The *k*_cat_ and *K*_M_ values were reported to be 101 ± 10 s^−1^ and 6.6 ± 1.1 mM H_2_O_2,_ respectively. This gives a specificity constant *k*_cat_/*K*_M_ of 1.5 × 10^4^ M^−1^ s^−1^ [106]. In contrast to the catalase-like activity, the dQH_2_ peroxidase reaction is promptly, but reversibly, inhibited by NO (Figure 6). This suggests that the heme *d* site is directly involved in the binding and reduction of H_2_O_2_ (Figure 7). The reaction is also inhibited by HQNO (50% inhibition is measured at about 10-15 μM HQNO), emphasizing that dQH_2_ injects electrons directly into the quinol binding site of cytochrome *bd*-I. The observed high rates of the reaction indicate that it may have physiological significance in *E. coli*.

## 6. ROS and Heme-Copper Oxidases

The main function of heme-copper respiratory oxidases in mitochondria and most bacteria is highly efficient energy conversion and generation of the membrane potential (the proton motive force) due to the redox energy of O_2_ reduction to water [115,116]. The unique ability of heme-copper oxidases to pump protons through the membrane determines their distinctive features: the presence of a special device for a redox-coupled proton pump and intra-protein proton-conducting pathways arranged in a special way [37]. Each of the single-electron steps in the catalytic cycle of COX during the O_2_ reduction in the BNC (heme *a*_3_/Cu_B_) is associated with the transfer of ~1 pumped proton through the membrane. The catalytic cycle of heme-copper oxidases is a highly coordinated system of individual electrogenic stages of electron transfer from cytochrome *c* on the P-side of the membrane and substrate protons on the N-side through the protein matrix to the BNC, as well as the transfer of pumped protons from the N-side of the membrane through temporary loading proton sites to the external water phase [32].

The BNC of COX is designed by nature to avoid, during the reduction of O_2_, producing of free forms of ROS, which would be released to the bulk phase. After binding of the oxygen molecule to heme *a*_3_ in the reduced BNC, the O-O bond is broken and four electrons are transferred to O_2_ in virtually one step. The heme *a*_3_ iron gives up two electrons and is oxidized to an oxidation state of +4, while Cu_B_ and the redox-active tyrosine residue give the other two electrons for complete reduction of the oxygen atoms to produce two molecules of water. The resulting P_M_ catalytic intermediate is homologous to compound I of peroxidases. The P_0_ compound corresponding to compound 0 in horseradish peroxidase with the bound primary H_2_O_2_ adduct of the heme moiety was not time-resolved in the case of COX of mitochondria and other heme-copper oxidases of the A family. P_M_ has an oxoferryl state of heme *a*_3_ with the oxidized tyrosine residue (the radical form) whose reduction by an electron from cytochrome *c* (the third electron in the COX catalytic cycle) and protonation of the hydroxyl bound to Cu_B_ lead to the F state. The F state is homologous to compound II of peroxidases. In the heme-copper oxidases of the B family, only the intermediate state P was kinetically resolved [117]. The intermediate state F was observed only in stationary measurements during prolonged incubation with excess H_2_O_2_ (for details, see [118]). For the heme-copper oxidases of the C family, only computer calculations were reported. According to these calculations, the P_M_ state is not energetically favorable and is not formed [119].

In addition to the main reaction, for COX from mitochondria, peroxidase-like and catalase-like activities were demonstrated. It was found that COX can catalyze the reduction of H_2_O_2_ in the presence of cytochrome *c*, i.e., cytochrome *c* peroxidase-like reaction [120]. The catalase-like activity (dismutation of H_2_O_2_) was observed initially by monitoring spectrophotometrically how the mitochondrial COX reduces the concentration of added H_2_O_2_ in the absence of an external electron donor [121]. This catalase-like activity of COX was described as dismutation of H_2_O_2_ with a turnover number of about 100 min^−1^. Recently, a second-order rate constant of 60–200 M^−1^·s^−1^ for the catalase-like activity of the bovine COX was obtained in more accurate measurements using an H_2_O_2_-sensitive electrode [122,123].

In the course of the reaction with H_2_O_2_, the BNC of COX goes through the same intermediates (P_M_ and F), which are resolved during the O_2_ reduction. The P_M_ and F intermediates of the mitochondrial COX (with different relative ratios) can be obtained in a steady state in the presence of H_2_O_2_. The pre-steady state measurements showed that the interaction of the BNC with two H_2_O_2_ molecules leads to the sequential formation of P_M_ and the reduction of P_M_ to F by the second H_2_O_2_ molecule with the production of O_2_^•−^ [124]. During the reaction of the mitochondrial COX with H_2_O_2_ at a high concentration, two molecules of H_2_O_2_ reduce the P_M_ state formed upon the binding of the first H_2_O_2_ to heme *a*_3_. Two molecules of O_2_^•−^ are formed in the BNC and undergo dismutation into the new H_2_O_2_ molecule [124]. At submillimolar concentrations of H_2_O_2_, its decomposition occurs at least at two sites: (i) the catalytic heme *a*_3_−Cu_B_ center where H_2_O_2_ is reduced to water via the P_M_ and F states, and (ii) the surface-exposed lipid-based radicals generated due to the migration of radicals formed initially in the catalytic heme *a*_3_−Cu_B_ center [125].

The mitochondrial COX can oxidize various aromatic compounds including some pharmacologically and physiologically active substances via the peroxidase mechanism [122]. Noticeably, the rates of both catalase-like and peroxidase-like activities of the mitochondrial COX are several orders of magnitude less than those for the true catalases and specific peroxidases (10^7^ M^−1^·s^−1^). Hence, against the background of the specialized enzymes designed to scavenge ROS, the “parasitic” reactions (peroxidase-like and catalase-like activities) of the mitochondrial COX can be characterized as side reactions. For this reason, they are unlikely to be of physiological significance in the ROS detoxification in mitochondria. However, COX is present at a high concentration in all tissues in the body, and often there are tissues, such as the myocardium, in which there is no peroxidase at all against the background of large numbers of mitochondria. Additionally, specific localization of the enzyme in the mitochondrial membrane promotes the accumulation of hydrophobic aromatic substances. Thus, the nonspecific peroxidation catalyzed by COX via the peroxidase mechanism should be taken into account in some cases (e.g., metabolism of hydrophobic medicinal or cardiotoxic compounds) [122]. It should be noted that cytochrome *c*, which possesses peroxidase-like activity, could protect against ROS production in mitochondria [126].

Even though in mitochondria the function of direct ROS detoxification, a kind of “manual” work, is performed very effectively by specialized enzymes (peroxidases, catalases, superoxide dismutase, and glutathione reductases), COX nevertheless participates in the control of ROS but at a higher level of organization, through an indirect mechanism of ROS regulation in which COX performs signaling, rather than a catalytic function. The mechanism of reversible “allosteric ATP-inhibition” of dimeric COX keeps the ROS production and heat generation low in mitochondria by maintaining low values for the mitochondrial inner membrane potential [127]. This ability of COX to prevent oxygen radical formation and cellular damage is canceled by increased intracellular calcium, as a consequence of stress, which dephosphorylates and monomerizes COX.

The decomposition of H_2_O_2_ by the prokaryotic *aa*_3_-type cytochrome *c* oxidases from *Rhodobacter sphaeroides* and *Paracoccus denitrificans* (homologous to the mitochondrial COX) occurs at a rate of ten or more times faster as compared to the enzyme from mitochondria (up to 2800 and 3300 M^−1^·s^−1^, respectively) [122,128]. In contrast to the bovine enzyme, the observed rate of H_2_O_2_ decomposition by the bacterial COXs is too high to be explained by the catalytic cleavage of H_2_O_2_ in the oxygen reducing center, since the rate of H_2_O_2_ binding to the BNC is significantly smaller (500–800 M^−1^·s^−1^) than the catalase-like activity. This may indicate the protective significance of these “parasitic” reactions in bacteria. There is reason to believe that the Mg ion located in the A family COXs near the proposed proton-releasing pathways (for references, see [129]) can be replaced by the Mn ion, depending on the environment in which the bacteria exist, and this ion can perform a catalytic function [122]. Meanwhile, the Mn ions are known to be part of the catalytic center of peroxidases and very good catalysts for the peroxidase reaction.

It is known that inhibition of mitochondrial respiration by NO (targeting COX) and its derivatives stimulates ROS and RNS production by mitochondria, which have signaling roles in the heart but may also contribute to cell death [130]. In contrast to the A family mitochondrial COX, which is inhibited by NO, the NO reductase activity is observed for the B family heme-copper oxidases, e.g., the *ba*_3_ oxidase from *Thermus thermophilus* [131]. It is suggested that this activity may be related to the higher Cu_B_ affinity of these enzymes for gaseous ligands. It is known that the activity of NO reductase, an enzyme related to heme-copper oxidases, provides resistance of some bacteria to the immune response of macrophages [132]. The presence of the NO reductase activity in prokaryotic heme-copper oxidases may provide pathogenic bacteria with the antioxidant capacity to protect against ROS and RNS in the course of an immune response and develop resistance against these harmful species.

Finally, in bacteria, the heme-copper oxidases of the C family (*cbb*_3_-type enzymes), which are expressed in low-oxygen environments, can also perform a protective function against ROS, and are in some cases very effective. The high O_2_ affinity cytochrome *cbb*_3_, along with the *bd* oxidase, plays an important role in the protection of O_2_-sensitive nitrogenase in *A. caulinodans* by quickly consuming O_2_. The *A. caulinodans* mutant strain devoid of both terminal oxidases is no longer capable of fixing N_2_ [84]. Akin to cytochrome *bd*, the *cbb*_3_-type oxidase is necessary to reduce the environmental O_2_ pressure before anaerobic photosynthesis. Accordingly, in contrast to the wild-type *R. gelatinosus* strain, the double mutant lacking both *cbb*_3_ and *bd* oxidases can initiate photosynthesis only in a deoxygenated medium [90]. The C family heme-copper oxidases have been much less studied than the oxidases of the other families. For the oxidases of the B and C families, variability in the stoichiometry of proton pumping was reported. How this could be related/correlated to their activity to be expressed under low O_2_ conditions, as well as to the ability to suppress ROS, remains to be elucidated.

## 7. Concluding Remarks

**Figure 8:**
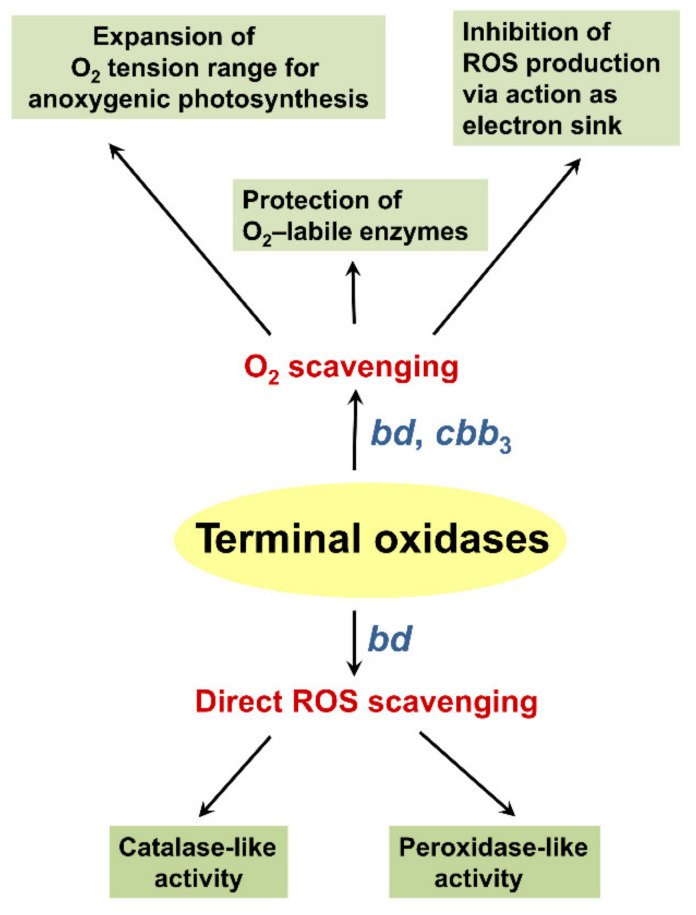
Overview of the proposed contribution of terminal oxidases to ROS defense mechanisms in bacteria.

Bacteria have evolved elaborate strategies to defend themselves from ROS and minimize oxidative damage. Many specialized detoxifying enzymes, such as superoxide dismutases, catalases, and peroxidases, have been extensively characterized. In this review, according to recent data, we report that terminal oxidases in bacterial respiratory chains may also play a protective role against ROS (Figure 8). Being efficient O_2_ scavengers, both copper-lacking cytochrome *bd* and the heme-copper oxidase *cbb*_3_ protect nitrogenase, the O_2_-labile enzyme complex responsible for catalyzing N_2_ fixation, from inactivation by O_2_, as documented in *A. caulinodans*, *A. vinelandii*, and *K. pneumoniae*. The *bd* and *cbb*_3_ oxidases also reduce the environmental O_2_ pressure, thereby expanding the physiological range of O_2_ tensions for the anoxygenic phototroph *R. gelatinosus*, which allows photosynthesis to start. The *bd*-type enzyme gives *B. fragilis* and *D. gigas*, classified as strict anaerobes, the ability to survive in low-oxygen environments. Furthermore, the *E. coli* cytochrome *bd*-I pulls electrons away from ROS-producing fumarate reductase, which leads to a reduced amount of ROS. Finally, cytochrome *bd*-I and cytochrome *bd*-II from *E. coli* may directly metabolize H_2_O_2_ through the catalase mechanism. The former cytochrome can apparently catalyze ROS removal through another mechanism as well, acting as a quinol peroxidase.

These relevant features of bacterial terminal oxidases may provide opportunities for biotechnological applications aimed at increasing O_2_ and ROS resistance in microbes and open up an attractive area of study for the development of novel antimicrobials to fight the increasingly serious threat of antibiotic resistance in pathogenic microorganisms.

## Abbreviations

BNC	binuclear center
CO	carbon monoxide
COX	cytochrome *c* oxidase
dQH_2_	decyl-ubiquinol
DTT	dithiothreitol
HQNO	2-*n*-heptyl 4-hydroxyquinoline-N-oxide
NO	nitric oxide
Q_1_	2,3-dimethoxy-5-methyl-6-(3-methyl-2-butenyl)-1,4-benzoquinone
